# The FLURESP European commission project: cost-effectiveness assessment of ten public health measures against influenza in Italy: is there an interest in COVID-19 pandemic?

**DOI:** 10.1186/s12962-023-00432-0

**Published:** 2023-05-15

**Authors:** Ariel Beresniak, Christian Napoli, John Oxford, Alejandra Daruich, Laurent Niddam, Gérard Duru, Alberto E. Tozzi, Marta Ciofi degli Atti, Danielle Dupont, Caterina Rizzo, Dominique Bremond-Gignac

**Affiliations:** 1 Data Mining International, Geneva Business Terminal, Route de Pré-Bois, 14, 1216 Geneva, Switzerland; 2 Department of Medical Surgical Sciences and Translational Medicine, Sapienza University of Rome, Rome, Italy; 3 Queen Mary College, London, UK; 4 University Hospital Necker-Enfants Malades, Assistance Publique-Hôpitaux de Paris, AP-HP, Paris University, Paris, France; 5 Research Centre Cordeliers, INSERM, UMRS1138, Team 17, Sorbonne Paris Cité University, Paris, France; 6 Niddam European Community Lawyer, Budapest, Hungary; 7 IRCCS, Bambino Gesù Children’s Hospital, Rome, Italy

**Keywords:** Human influenza, Cost-Effectiveness, Pandemics, Emergency preparedness, COVID-19, SARS-CoV-2

## Abstract

**Background:**

The FLURESP project is a public health research funded by the European Commission, with the objective to design a methodological framework to assess the cost-effectiveness of existing public health measures against human influenza pandemics. A dataset has been specifically collected in the frame of the Italian health system. As most of interventions against human influenza are relavant against other respiratory diseases pandemics, potential interests in COVID-19 are discussed.

**Methods:**

Ten public health measures against human influenza pandemics pandemic were selected to be also relevant to other respiratory virus pandemics such as COVID 19: individual (hand washing, using masks), border control (quarantine, fever screening, border closure), community infection (school closure, class dismissal, social distancing, limitation of public transport), reduction of secondary infections (implementation of antibiotic therapy guidelines), pneumococcal vaccination for at-risk people, development of Intensive Care Unit (ICU) capacity, implementation of life support equipments in ICU, screening interventions, vaccination programs targeting health professional and targeting general population.

**Results:**

Using mortality reduction as effectiveness criteria, the most cost-effective strategies are “reduction of secondary infections” and “implementation of life support equipment in ICU”. The least cost-effective option whatever the level of pandemic events are screening interventions and mass vaccination.

**Conclusions:**

A number of intervention strategies against human influenza pandemics appears relevant against every respiratory virus, including the COVID-19 event. Measures against pandemics should be considered according to their expected effectiveness but also their costs for the society because they impose substantial burden to the population, confirming the interest of considering cost-effectiveness of public health measures to enlighten decision making.

## Introduction

A number of viruses infecting a spectrum of warm-blooded animals, including pigs, horses, birds, bats can switch hosts to form new lineages in novel hosts, leading to potential human to human contaminations and global pandemics. In 2009, the spread of a novel H1N1 strain of human Influenza attained pandemic proportions. In response, developed nations initiated in a very short time frame a battery of public health measures [1]. In June 2013, the World Health Organization (WHO) published the Pandemic Influenza Risk Management Interim Guidance to mitigate the risks of pandemic threats [2]. Although this guidance has socio-economic implications [3], cost-effectiveness evaluations were not clearly considered by decision makers, very probably because of the challenges in measuring and communicating about the impact of public health interventions. These considerations led to the launch of the FLURESP project, a 42 months public health research project funded by the European Commission, with the objective to design a practical but robust methodological approach for comparing the cost-effectiveness of public health measures against pandemics in four target countries: Italy, France, Poland and Romania [4, 5].

Ten years after the H1N1 event, a new pandemic of another respiratory virus appeared. This worldwide pandemic of coronavirus disease 2019 (COVID-19) is caused by a severe acute respiratory syndrome coronavirus 2 (SARS-CoV-2) and was first confirmed to have spread to Italy on January 30 2020, when two Chinese tourists in Rome tested positive for the virus. By the beginning of March 2020, the virus had spread to all regions of Italy [6]. Public health interventions implemented by the Italian government started with the cancellation of all flights, imposed social distancing measures and population lock down. In the context of this new global pandemic, and considering that in the last decades, several emerging and re-emerging infectious diseases (such as SARS, MERS, avian influenza, 2009-influenza pandemic and Ebola) have had an important impact on preparedness plans in different settings, it is then appropriate to ask which public health interventions against human influenza would have been most relevant against COVID-19, and which public health interventions used against COVID-19 might have already been assessed against influenza pandemics. Considering that decision makers in Europe had to react against a new pandemic to address a public health emergency, robust and transparent cost-effectiveness studies comparing a large spectrum of public health measures is needed to assist optimal decision-making. Beyond clinical research studies assessing the efficacy and safety of vaccines and antiviral medications, cost-effectiveness studies comparing the value of different public health interventions using similar effectiveness criteria are still limited in the frame of the COVID-19 pandemic [7–9]. For these reasons stakeholders and policy makers have expressed the need and high expectations for the development of transparent cost-effectiveness studies to assess and compare different public health measures against pandemics.

The World Health Organization (WHO) published the Pandemic Influenza Risk Management Interim Guidance [2] including some socio-economic considerations excluding cost-effectiveness evaluations, very probably because of the challenges in measuring the impact of public health interventions and the small number of comparative studies of public health responses[4]. Pérez Velasco et al. [10] published a systematic review identifying 44 economic evaluations in pandemic preparedness strategies studies of which 34 (77%) only focused on therapeutic interventions [11–20]. Only four studies (9%) focused solely on non-therapeutic interventions (school closures, air travel restrictions, sick leave authorizations, use of face masks) [21–23]. Thus, our litterature review confirms an important intervention selection bias to the detriment of non- therapeutic interventions [24, 25].

These considerations existed before the COVID-19 pandemic and led to the launch of the FLURESP project, a public health research funded by the European Commission, with the objective to design a methodological framework to assess and compare the cost-effectiveness of existing public health measures against human influenza pandemics in four target countries: France, Italy, Poland and Romania [4]. This paper presents the results relevant to the Italian health system using existing data sources specifically investigated for this purpose.

## Materials and methods

### Scope of the research

Whilst therapeutic interventions against influenza pandemic have been well documented, other (non therapeutic) public health measures have rarely been evaluated and compared [26]. This is also the case for new COVID-19 pandemic. Then the real value and impact of public health measures on the outbreak is poorly understood. The aim of the FLURESP study was to compare costs and effectiveness of public health measures against human influenza pandemic, which allow to discuss potential interest for the COVID-19 pandemic. The scope of the study is limited to comparing costs, effectiveness and cost-effectiveness of ten public health interventions against human influenza pandemics.

### Research design

A list of public health measures against influenza was extracted from preparedness plans [27, 28]. Interventions were then clustered into 6 categories (Table 1), representing 10 public health measures against both influenza and other respiratory virus pandemics (Individual measures, Border control, Community infection control measures, Reduction of secondary infections, Pneumococcal vaccination, development of Intensive Care Unit capacity, Implementation of life support equipment).Screening interventions and Immunization programs.

**Table 1:** List of response strategies, public health measures and scope of interventions

Response strategy	Public health measures	Scope of interventions
Individual disease transmission	Individual measures	Hand washing, mask wearing, respiratory etiquette
Societal Interventions	Border control	Quarantine, fever screening, border closure
	Community infection control measures	School closure, class dismissal, staggering, masks in public areas, social distancing, limitation of public transports, etc
Reducing secondary infections	Reduction of secondary infections	Implementation of guidelines about antibiotic therapy
	Pneumococcal vaccination	People at risk
Level of care	Development of new ICU capacity	New ICU capacity
	Implementation of life support equipment	Ventilators, Extracorporeal Membrane Oxygenation equipment
Screening	Screening interventions	Virus testing
Immunization	Vaccination program targeting selected populations	Vaccination of Health professionals Vaccination of general population

ICU: intensive care units

Six pandemic scenarios were assessed according to a new typology of 4 parameters specifically defined in the frame of the FLURESP European project, as published in by Napoli et al. [5]:

1. Clinical Attack Rate (CAR): proportion of the population infected and with symptoms.
2. Case Fatality Rate (CFR): proportion of infected individuals who die from the infection and its complications.
3. Hospital Admission Rate (HAR): proportion of the population admitted to hospitals for confirmed influenza independently of the presence of complications
4. Intensive Care Usage (ICUS): proportion of confirmed hospitalizations that need to be treated in Intensive Care Units for complications.

Combining these 4 parameters generates 6 pandemic scenarios (Table 2):

**Table 2:** Presentation of the six epidemic scenarios (A, B, C, D, E) identified by the combination of selected parameters published in Eurosurveillance, from Napoli et al. [5]

		Pandemic scenario					
		A: Seasonal like %	B: 2009 pandemic like %	C: Community risk /low virulence%	D: Community risk /high virulence %b	E: High risk groups /age classes %	F: Major event %
Transmission	CAR	0–5	5–10	10–25	10–25	10–25	25–35
Virulence	CFR	0–0.01	0–0.01	0–0.01	0.01–0.05	0.05–0.8	0.8–2.5
Medical ressources utilization	HAR	0–0.02	0.2–2	0–0.02	0.2–2	0.2–2	2–4
	ICUS	0–0.01	0–0.01	0–0.01	2.5–5	2.5–5	5–35

Scenario A ("Seasonal like"), Scenario B ("2009 pandemic like"), Scenario C ("Community risk, low virulence"), Scenario D ("Community risk, high virulence"), Scenario E ("High risk groups") and Scenario F ("Major event") [5].

The target of population of this economic evaluation consists of the general population in Italy. The time horizon of the evaluation is three quarters (9 months) which corresponds to the duration of previous pandemics due to respiratory viruses [29, 30].

### Research approach

The research approach is based on the selection of one relevant effectiveness criteria deemed appropriate for the eight public health measures. Relevant to human influenza, and considering that the most important complications of COVID-19 are respiratory distress and death, the outcome “achieving mortality reduction higher or equal to 40%” was selected as a meaningful “therapeutic success” outcome as in previously published assessments of public health interventions against influenza pandemics[4]. This outcome enabled the use of a dichotomous variable for achieving either "Success or No Success" when assessing each public health measure. The selection of a threshold allowed to transform quantitative variables (mortality, morbidity) in binary variables (Yes/No). However, no specific indications about potential suitable mortality or morbidity thresholds have been suggested in the literature. This is the reason why the proposed threshold has been estimated as “expert opinion” by consensus by the study experts which were all very experienced in public health programs. Success probabilities were sampled from a uniform distribution between minimum and maximum values (Table 3) according to data collected during the A/H1N1 2009 pandemic [4].

**Table 3:** Estimated probabilities for effectiveness criteria “Achieving mortality reduction  ≥ 40%” [4]

Individual measures	1–5%
Border control	1–5%
Community infection control measures	1–5%
Reduction of secondary infections	1–5%
Pneumococcal vaccination	1–5%
Development of new ICU capacity	1–95%
Implementation of Life support equipment	90–95%
Screening interventions	1–5%
Vaccination program of Health Professionals	1–28%
Vaccination program of General population	25–72%

ICU: intensive care unit

Direct costs for each response strategy were expressed in Euros and included costs of the public health intervention and those of specific communication programs at the Italian national level for each epidemic scenario.

### Methods

The method followed a two-step approach: assessment of cost distribution for each strategy and calculation of cost-effectiveness ratios based on published success rates [4]. The perspective of the study is the Italian health system, while direct costs for each response strategy are expressed in Euros (2017).

The variability of the costs of each public health measure has been considered by programming distribution costs according to a uniform distribution between minimum and maximum values for each of the 10 public health measures, according to the 6 pandemic scenarios (Table 4).

**Table 4:** Costs table (in Million €) of the 8 interventions according to the 6 categories of pandemic scenarios

	Pandemic scenario											
	A		B		C		D		E		F	
	Min.	Max.	Min.	Max.	Min.	Max.	Min.	Max.	Min.	Max.	Min.	Max.
Individual measures	1.6	11	1.6	11	1.6	11	1.6	11	1.6	11	1.6	11
Border control	1.75	22.65	4.22	37.11	6.75	80.47	6.75	80.47	6.75	80.47	19.4	80.47
Community infection control measures	400.5	10,011	400.5	10,011	400.5	10,011	400.5	10,011	400.5	10,011	400.5	10,011
Reduction of secondary infections	0.14	1.4	0.14	1.4	0.14	1.4	0.14	1.4	0.14	1.4	0.14	1.4
Pneumococcal vaccination	3	24	3	24	3	24	3	24	3	24	3	24
Development of new ICU capacity	0.1	1.42	0.1	1.85	0.15	3.12	127.88	1065.88	127.88	1065.88	894.6	7455.22
Life support equipments	0.1	1.13	0.1	1.26	0.13	1.66	8.46	335.67	8.46	335.67	58.66	2343.75
Screening intervention	720.6	52,800,077	720.6	52,800,077	2352.96	22,418.50	720.6	52,800,077	720.6	52,800,077	720.6	52,800,077
Vaccination program of Health Professionals	1.3	18.4	1.3	18.4	1.3	18.4	1.3	18.4	1.3	18.4	1.3	18.4
Vaccination program of General population	2483.2	2487.8	2483.2	2487.8	2483.2	2487.8	2483.2	2487.8	2483.2	2487.8	2483.2	2487.8

Costing data in Italy were collected using an ad-hoc cost survey conducted in the framework of the FLURESP European project [4] using unpublished ministry of health reports. In the absence of costing data being publicly available, relevant costing information was collected through direct interviews with health programs supervisors and stakeholders (i.e.: manufacturers of ventilators and ECMO). Direct costs included supply chain costs, service delivery costs and communication costs. Costs uncertainty has been taken into account by managing their variability for each public health measure. Then costs were programmed according to a uniform distribution between minimum and maximum values for each public health measures, according to the 6 pandemic scenarios (Table 4). Because of the short time horizon of the evaluation, no discount rate was applied.

Public health data being often unreliable, traditional sensitivity analyses are cumbersome when more than two values vary concurrently. Considering that the uncertainty of each value can be assumed to possess a probability distribution, probabilistic sensitivity analyses (PSA) allow that all potential values are considered simultaneously [31]. In order to take into account the uncertainty about the cost and effectiveness of each strategy, PSA have been carried out considering that the cost of each strategy is distributed according to uniform distribution over the interval [Cost_min_-Cost_max_], and that the effectiveness was also uniformly distributed over the interval [Eff_min_-Eff_max_]. Distribution types such as triangular, normal, beta or discrete would have been relevant for estimating specific variables. However in the absence of key distribution parameters such as mean, mode, standard deviation, etc. and knowing minimum and maximum estimates, it was possible to only simulate uniform distributions. This also allow to not have to assume any hypothetical normal distributions, while Monte-Carlo simulations have been carried out as PSA. 10,000 Monte Carlo simulations of cost and effectiveness to provide an empirical distribution of cost-effectiveness and the average noted ACER (Average Cost-Effectiveness Ratio), expressed as costs per success. For this purpose, the analyses computed all potential values of the numerator and denominator distributions [32, 33].

## Results

Considering the success outcome of achieving mortality reduction  ≥ 40%, ACER are expressed as "Costs to achieve a mortality reduction  ≥ 40%". ACER of the 8 interventions according to the 6 pandemic scenarios are presented in Table 5.

**Table 5:** Cost-Effectiveness ratios in costs (M€) /success (achieving a mortality reduction ≥ 40%)

	**Pandemic scenario**					
	**A**	**B**	**C**	**D**	**E**	**F**
Individual measures	253.67	253.67	253.67	253.67	253.67	253.67
Border control	490.24	831.86	1755.03	1755.03	1755.03	2012.15
Community infection control measures	209409.26	209409.26	209409.26	209409.26	209409.26	209409.26
Reduction of secondary infections	30.99	30.99	30.99	30.99	30.99	30.99
Pneumococcal vaccination	543.49	543.49	543.49	543.49	543.49	543.49
Development of new ICU capacity	3.68	3.68	7.91	2893.38	2893.38	20237.91
Life support equipment	0.66	0.73	0.97	185.57	185.57	1295.45
Screening Interventions	1′061′547′989	1′061′547′989	1′061′547′989	1′061′547′989	1′061′547′989	1′061′547′989
Vaccination health professionals	629′540′680	629′540′680	629′540′680	629′540′680	629′540′680	629′540′680
Vaccination general population	1′409′493′454	1′409′493′45	1′409′493′45	1′409′493′45	1′409′493′45	1′409′493′45

*ICU: Intensive Care Units*

The FLURESP study showed that the three most cost-effective public health strategies against human influenza using a reduction of mortality as effectiveness criterion were the “reduction of secondary infections” and “implementation of life support equipment in ICU”.

The less cost-effective option using reduction of mortality as effectiveness criterion was "screening measures" and mass vaccination of general population, whatever the epidemic scenarios with estimated costs respectively of 1061.5 and 1409.4 million euros for achieving a reduction of  ≥ 40% of mortality rate.

In the more severe epidemic scenarios D-E-F, the most cost-effective public health measure were “reduction of secondary infections” using antibiotic guidelines (30.99 million euros per success, whatever the epidemic scenario), followed by “New life support equipment” (185.57 Million euros per success for epidemic scenarios D and E, and 1295.45 million euros per success for the major event scenario F).

Individual measures (hand washing, etc.) appears a cost-effective option (253.67 million euros per success for any epidemic scenarios), particularly in severe epidemic scenarios when the costs of other public health alternatives are more expensive.

Cost-effectiveness ratios of the measure “Border control” increase from 490.24 million euros for epidemic scenario A to 2012.15 million Euros to the major event pandemic scenario F. Then for epidemic scenario A, the “Border Control” intervention is less cost-effective than “Individual measures”, “Reduction of secondary infections”, “Development of new ICU capacity” and “Life support equipment” but more cost-effective than “Community infection control measures”, “Pneumococcal vaccination” and “Screening intervention”.

The strategy “Community infection control measures”, including social distancing measures such as school closure, masks in public areas, limitation of public transports, etc., appears the second less cost-effective option (209,409.26 million euros per success, whatever epidemic scenarios).

In contrast, the most cost-effective options is the “life support equipment” applied to epidemic scenario A, B, C with estimated ACER of 660,000 euros for epidemic scenario A, 730,000 euros per success for epidemic Scenario B, 970,000 euros per success for epidemic scenario C.

### Probability graphs

**Fig. 1:**
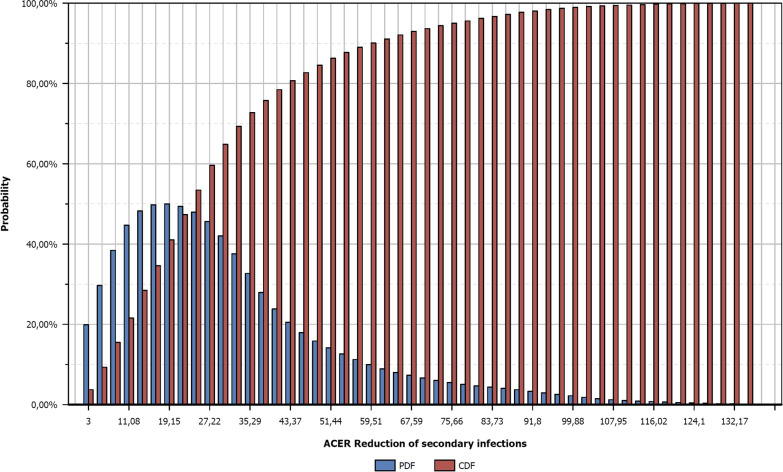
Probability Distribution Function (PDF) and Cumulative Distribution Function (CDF) of the intervention "Reduction of secondary infection" in the frame of any pandemic scenario. Blue bars represent the ACER distribution and red bars represent the cumulative distribution of ACER. For example there is a probability up to 85% to cost less than 186 Million Euros

**Fig. 2:**
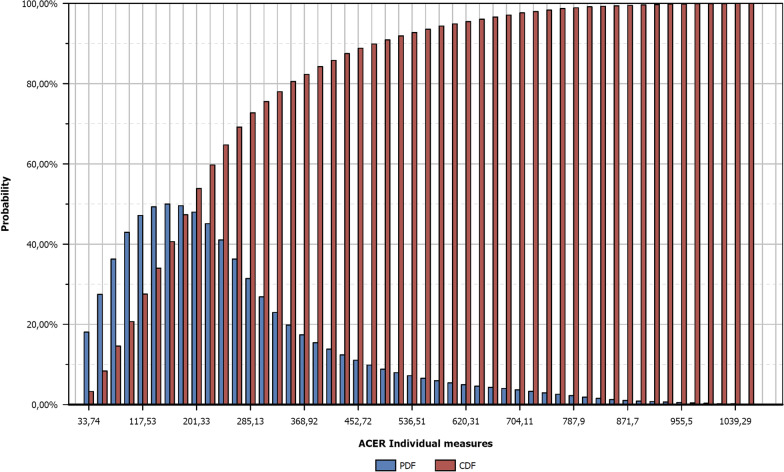
Probability Distribution Function (PDF) and Cumulative Distribution Function (CDF) of the intervention "Individual measures" in the frame of any pandemic scenarios. Blue bars represent the ACER distribution and red bars represent the cumulative distribution of ACER. For example, there is a probability up to 80% to cost less than 347 Million Euros

PSA on the conducted analyses allow to generated probability graphs. As it is not possible to present such a diagram for each of the 60 case studies (10 public health measures according to 6 pandemic scenarios), the 2 following figures are presented as examples. Figure 1 represents the intervention "Reduction of secondary infections " during all types of pandemics and Fig. 2 represents the intervention "Individual measures" during all types of pandemics.The blue bars represent the distributions (PDF) of the cost-effectiveness ratios and the red bars represents the cumulative probability function (CPF).

## Discussion

Among the number of cost-effectiveness ratios generated, one of the most interesting results suggests that interventions such as mass vaccination, screening, border control or individual measures would not be cost-effective measures when implemented separately, particularly in severe scenarios of pandemics. However, considering that no other mitigation measures are available to date, even if such strategies would not be cost-effective, screening, border control and individual measures have been widely implemented during the major COVID-19 events in Italy and in Europe. The main explanations are that not only the cost-effectiveness assessments of these public health measures were not available at that time to assist decision-making, but also, the economic impacts and economic arbitrations were not the priority of policy makers given the perceived urgency of such new pandemic. Immunization strategies of either health professionals or general population does not seem to be cost-effective strategies, suggesting that such interventions would impact more positively disease morbidity than disease mortality.

Another interesting results concerns the good cost-effectiveness ratios based on mortality outcomes of the implementation of new life support equipment (such as ventilators and ECMO). Since its use in the 1950s in operating rooms, life support equipment is becoming a promising rescue strategy for cardiopulmonary arrest in ICU. With expanding potential indications to acute respiratory distress syndrome in the frame of severe influenza (or COVID-19), there is significant interest in the early application of life support equipment in the Emergency Departments during pandemics [34].

The H1N1 pandemic brought life support equipment technology into the light with excellent results of reducing mortality caused by respiratory distress, which would explain the favourable ACER. These results are quite robust because of the wide distribution range of potential costs of implementing such equipment as assessed in the Monte Carlo simulations (between 0.1 and 1.13 M€ for pandemic scenarios types A-B-C, between 8.46 and 335.67 M€ for epidemic scenarios D-E and between 58.66 and 2343.75 M€ for major event epidemic scenario D) (Table 4).

These economic estimates regarding life support equipment versus other interventions should contribute to deploying the necessary life support material in the Italian national territories in a timely manner. Interestingly, this equipment could be also used to treat a growing number of other potential indications such as acute hypoxemic respiratory failure, cardiac arrest or cardiogenic shock related to this new pandemic. Continuing research in Public Health mitigation and containment measures for pandemics including economic evaluations would likely spur further expansion of life support equipment during influenza pandemics [35].

Comparing different public health interventions has been possible by selecting common and meaningful effectiveness outcomes based on morbidity and mortality reductions suitable to each intervention. Comparisons of such different public health interventions against pandemics is not commonly undertaken for several reasons: the lack of comparative studies of public health measures, the cultural variability about preventive strategies, the absence of international consensus on optimal strategies to consider, the epidemiological conditions for switching to an alternative measure, and the precise switching strategy in case of insufficient response to a previous intervention.

As for every cost-effectiveness study, this research is subject of various sources of bias concerning costs and effectiveness estimates. Bias is defined as a systematic difference in an observed measurement from the true value. For example, hidden costs such as cost of lost work days could cause systematic measurement errors. In the context of public health interventions assessment study, bias occurs when the overall estimates systematically deviate from the real value. Bias and imprecision are both sources of variation that can cause an estimate to differ from the true value but are also extremely difficult to control when assessing public health interventions [36]. The lack of appraisal of outcome reporting bias in the public health literature may be due to the wide range of study designs that are appropriate to assess complex public health interventions and the lack of randomization [37, 38]. The resulting diversity in reporting practice and ethical issues raised by any randomized designs for assessing public health measures makes the assessment of outcome reporting bias considerably more difficult [39], even if many evidence confirms that outcome reporting bias also exists in state of the art randomized controlled trials. Whatever, it is acceptable to assume that directs costs are a part of total costs of one public health measure, and total costs are systematically much higher than direct costs only as it also includes indirect, intangible, and inter-sectoral costs. Because no robust sources have been identified reporting indirect and inter-sectorals costs, only direct costs have been considered in this study, which is the main limitation.

The feasibility of such studies is also questioned because conducting comparative studies with potential suboptimal prevention strategies would likely raise ethical issues during a major outbreak. Furthermore, the prohibitive cost of conducting multi-arm trials to assess and compare a combination of various public health interventions used concurrently and/or in a sequential manner make such an experiment totally impractical. One alternative includes conducting simulations using advanced modelling approaches, as carried out in the frame of the FLURESP project [4, 40]. This include complex combinations of public health interventions was performed and compared in a nine month timeframe divided into 3 quarters Q1 (announcement of the pandemic), Q2 (Cases detected in the country but epidemic threshold not yet achieved) and Q3 (Pandemic wave in the country). The following sequence of interventions was simulated for testing the feasibility of the approach: Q1: "Border control"; Q2: "Reduction of secondary infections" + "Pneumococcal vaccination" + "Screening intervention"; Q3: "Vaccination program of health professionals” + "Vaccination program of general population”. Even if the methodological approach would allow to calculate such simulations, no effectiveness data exist for being able to populate and simulating such combined scenarios, which should be considered only theoretical at this stage.

The main limitation of this study mirrors the limitation and the quality of the data, especially when data have not been specifically generated in the frame of this study. Using existing data from different sources directly impacts the key findings. Whatever, it should be noted that consistant cost-effectiveness results have been established in 3 other health systems (France, Romania, Poland) using the similar methodology and different local data sources. As for any economic evaluation studies, more precise and robust national results should be generated from high quality specific data collection.

Importantly, many published “cost-effectiveness” analyses used the synthetic indicator “Quality Adjusted Life Years” (QALY) as a standard outcome for comparing interventions [41–43]. This approach assesses utility measures of patient preferences calibrated between 0 (death) and 1 (full health). Technically entitled "Cost-Utility Analyses" this outcome is subject of an active methodological controversy in health economics. Both the scientific community and decision makers must be aware of this methodological debate in order to better understand why numerous publications present cost-utility analyses expressed in "costs per QALY" under the umbrella of “cost-effectiveness” analyses, without distinguishing that they consist in fact of two different methods, which are not equivalent, and not interchangeable.

The fact that effectiveness criteria based on mortality reduction selected for this project are clinically meaningful and relevant to all kinds of interventions is a major contribution for decision-making purposes. Selecting objective and consistent public health outcomes allows the performance of a given intervention to be assessed and expressed more accurately and compared across different strategies within various population groups. It is the reason why this study used a public-health-meaningful outcome such as "success rate" (achieving a mortality reduction of 40%) leading to cost-effectiveness ratios expressed in "costs per success". Of course, the selected thresholds of 40% reduction could be challenged, but could also be changed and adapted according to health authorities’ priorities. Another potential limitation of these results is that performance effectiveness values have been derived from published data concerning the French Health System [4]. Total costs and performance of public health interventions from country to country are rarely published in a consistent way, requiring specific surveys to be carried out in each target country. For example, basic epidemiological indicators such as morbidity and mortality indicators are collected and recorded differently among countries.

An additional limitation is that reported cost-effectiveness ratios present only direct costs. Indirect costs (lost productivity, absenteeism, etc.) appear to be important factors to consider in public health interventions against pandemics [44]. Furthermore, cost-effectiveness studies should also take into account the long term health impact, such as health losses and costs associated with untreated chronic conditions, as well as the broader socio-economic impact, including mental health and lost productivity [9, 45, 46], which were driver costs during the COVID pandemic. The lack of standardization and high variability of indirect costs however make national and international comparisons difficult. A primary reason why this study applied a narrow societal perspective is the methodological challenges associated with capturing these wider costs such as unavailable data. The identification and valuation of indirect costs such as intersectoral costs in economic is recognized as one of the main methodological challenges when assessing public health interventions [47]. Despite methodological difficulties, the narrow societal perspective of this study is recognized and justified for feasibility reasons. Concerning the generalizability of the cost-effectiveness results focused on the Italian health system, similar results have been generated in three other health systems: France [4], Romania and Poland. The results suggest that most cost-effective public health measures applied in one European country would likely be also be cost-effective in other European countries.

As for any economic evaluation studies however, precise national results should be generated from a robust dedicated data collection carried out in the relevant health systems. Furthermore, national data are also sensitive with time and such economic evaluation of public health interventions should be organized on a regular basis, ideally after each pandemic.

Then further cost-effectiveness studies should take into account the long term health impact, such as health losses and costs associated with untreated chronic conditions, as well as the broader socio-economic impact, including mental health and lost productivity [9, 45, 46].

Because various public health measures implemented during human influenza pandemics and the COVID-19 pandemic in Italy are practically the same, the results generated originally to assess interventions against various scenarios of influenza pandemics appear potentially relevant to any other respiratory viruses pandemics in general, including the COVID-19 pandemic. However novel public health measures have been experimented for the first time during the COVID-19 pandemic, such as lock down, curfews and vaccination passports. As these new measures were not included in any existing preparedness plans against Human influenza pandemic, they have not been assessed in the frame of the FLURESP project which then is unable to provide any information about the costs and performance of such measures.

The main implications of the FLURESP initiative are the following:

- The methodology confirms that it is possible to compare various interventions (therapeutic or non-therapeutic), using common and meaningful public health criteria
- The FLURESP project provides a framework for assessing public health measures, considering that most of public health interventions implemented during the COVID-19 pandemic have not been previously evaluated in term of costs and effectiveness
- The results led to guidelines for reducing pandemic mortality by ranking public measures by level of cost-effectiveness.

## Conclusions

The results of the FLURESP European project confirm that intervention strategies against outbreaks and pandemics, including non-pharmaceutical interventions, can impose a substantial economic burden, suggesting a need to develop such methodological approaches across countries. In allowing actions to be prioritized, it is expected that Cost-Effectiveness information would have increasing direct public health impact. Therefore, such assessments should be generated by country to enrich preparedness activities for various types of pandemics, including human influenza and other respiratory diseases such as COVID. This ambitious study is the very first to compare a large set of public health measures according to performance and costs, and should be considered as a positive feasibility study to be carried out on a regular basis. If many important data such as interventions effectiveness and costs are not collected in a routine way, there is no doubt that future preparedness strategies ought to be progressively enriched by cost-effectiveness considerations concerning a wide spectrum of relevant public health interventions. This will lead to the development of new guidelines and recommendations about how best to combat pandemics for maximizing the number of lives saved, as well as optimizing health outcomes and resource allocation.

## Data Availability

Relevant data are included in the article tables.
